# Positive association between body height and breast cancer prevalence: a retrospective study with 135,741 women in Germany

**DOI:** 10.1007/s10549-022-06730-0

**Published:** 2022-09-10

**Authors:** Niklas Gremke, Sebastian Griewing, Matthias Kalder, Karel Kostev

**Affiliations:** 1 Department of Gynecology and Obstetrics, University Hospital Marburg, Philipps-University Marburg, Baldingerstraße, 35043 Marburg, Germany; 2 Epidemiology, IQVIA, Frankfurt, Germany

**Keywords:** Breast cancer prevalence, Body height, Non-modifiable risk factors, Germany

## Abstract

**Purpose:**

The aim of this study was to analyze the prevalence of breast cancer in relation to body height and to investigate associations between body height and breast cancer in Germany.

**Methods:**

This retrospective cohort study included 135,741 adult women followed in one of 161 gynecology practices in Germany between January 2019 and December 2021. The 3 year prevalence of breast cancer (ICD-10: C50) during the study period was shown in relation to body height, which was included in this study as a five-category variable for women: ≤ 160 cm, 161–165 cm, 166–170 cm, 171–175 cm, > 175 cm. The associations between height and breast cancer were analyzed using logistic regression models adjusted for age and BMI.

**Results:**

The prevalence of breast cancer increased from 5.1% in women ≤ 160 cm to 6.8% in women > 175 cm in the age group 51–60, and from 9.2% in women ≤ 160 cm to 12.2% in women 171–175 cm in the age group > 60 years. The OR for breast cancer was 1.18 (95% CI 1.12–1.24) for every 10 cm increase in height. Compared to height ≤ 160 cm, the OR for height 166–170 cm was 1.26 (1.15–1.39), for 171–175 cm 1.43 (1.27–1.61), and for > 175 cm 1.49 (1.28–1.74).

**Conclusion:**

The results of this study suggest that greater body height in women is significantly related to an increased breast cancer risk.

## Introduction

Female breast cancer (BC) is the most commonly diagnosed cancer worldwide with approximately 2,3 million new cases per year [1]. Recent epidemiological data reveal an incidence of approximately 69,000 BC cases per year in Germany and BC is therefore also considered the most common cancer type in women in Germany [2]. However, there are numerous risk factors that can influence the odds of developing BC. These can be divided into modifiable (e.g., drinking alcohol, being overweight, not being physically active) and non-modifiable (e.g., older age, female sex, genetic mutations, height) [3]. Significantly, decades of epidemiological studies have focused largely on the association between overweight and BC, while the effect of height on BC risk has received far less attention [4–7]. In the past, a number of publications have shown that non-modifiable anthropometric factors such as height can influence the risk for several cancer sites such that tall people have an increased risk of cancer, although the results are inconsistent to some extent [4, 8–10]. In a large prospective UK cohort study including 1,297,124 women without previous cancer, subjects were followed up for cancer incidence and other confounding and modifying factors. In this study it turned out that the relative risk (RR) adjusted for multiple variables such as BMI and socioeconomic status for cancer overall was 1.16 (95% CI 1.14–1.17; *p* < 0.0001) for every 10 cm increase in height. In total, the height-related RRs increased significantly for ten assessed cancer sites (e.g., malignant melanoma, breast cancer, ovarian cancer, and endometrial cancer) [11].

However, there is a lack of evidence with regard to the relationship between body height and BC risk in Germany. Given that the range of height in each population is relatively small, large patient cohorts are needed to obtain reliable results. In response to this need, we report here a large retrospective study with 135,741 women in Germany followed in one of 161 gynecology practices in Germany between January 2019 and December 2021 to analyze the prevalence of BC in relation to body height and to investigate associations between height and BC adjusted for age and BMI.

## Methods

### Database

This study used data from the Disease Analyzer database (IQVIA). This database has already been described extensively in the literature [12]. To summarize, the Disease Analyzer database contains demographic, diagnosis, and prescription data from patients followed in general and specialized practices in Germany. Practices are selected for inclusion in the database based on multiple factors (i.e., specialty group, community size category, and German federal state), and the database is composed of around 3–5% of all practices in Germany. Diagnosis and prescription data are coded using the International Classification of Diseases, 10th revision (ICD-10), and the Anatomical Classification of Pharmaceutical Products of the European Pharmaceutical Marketing Research Association (EphMRA), respectively. It has previously been shown that the panel of practices included in the Disease Analyzer database is representative of general and specialized practices in Germany [12]. Finally, this database has already been used in previous studies focusing on BC [13, 14].

### Study population

This retrospective cohort study included 135,741 adult women followed in 161 gynecology practices in Germany between January 2019 and December 2021. The only inclusion criterion was at least one documented height value during this period. Height values were available for 135,741 (19.3%) out of 702,475 women.

### Study outcomes and variables

The outcome of the study was the prevalence of BC (ICD-10: C50) diagnoses during the study period as a function of height. Height was included in this study as a five-category variable for women: ≤ 160 cm, 161–165 cm, 166–170 cm, 171–175 cm, > 175 cm.

### Statistical analyses

Age at first visit in 2019–2021 was compared between height categories. As there was a strong relationship between height and age (taller women were younger), all analyses were either performed by age group or adjusted for age. First, the 3 years prevalence of BC was descriptively shown. Then, the associations between height and BC were analyzed using logistic regression models adjusted for age and BMI. The results of the regression analyses are displayed as odds ratios (ORs) and 95% confidence intervals (95% CI). In the first model, ORs showed the risk increase for each height category compared to ≤ 160 cm. In the second model, ORs showed how the risk of BC increased for every 10 cm increase in height. *P*-values lower than 0.05 were considered statistically significant. Analyses were conducted with SAS 9.4 (SAS Institute, Cary, US).

## Results

**Fig. 1:**
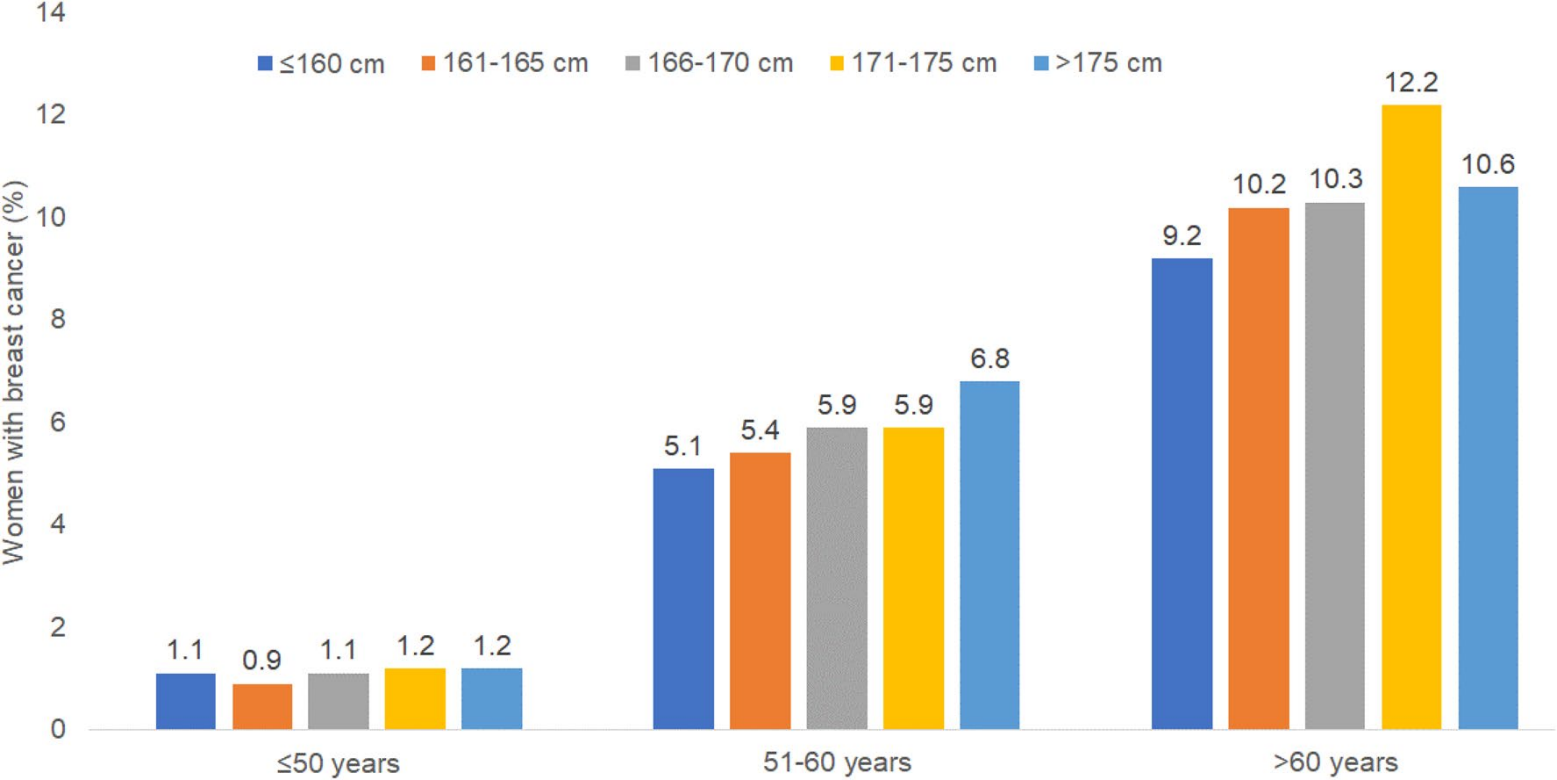
Prevalence of BC by age and height category

This study included 135,741 women with an average age of 39.8 years (SD 15.2). The average body height was 166.4 cm and the average BMI was 26.0 kg/m^2^ (Table 1). Figure 1 shows the prevalence of BC by age group and height category. The prevalence increased from 5.1% in women ≤ 160 cm to 6.8% in women > 175 cm in the age group 51–60, and from 9.2% in women ≤ 160 cm to 12.2% in women 171–175 cm in the age group > 60 years. The results of the age- and BMI-adjusted logistic regression analyses are displayed in Table 2. The OR for BC was 1.18 (95% CI 1.12–1.24) for every 10 cm increase in height. Compared to height ≤ 160 cm, the OR for height 166–170 cm was 1.26 (1.15–1.39), for 171–175 cm 1.43 (1.27–1.61), and for > 175 cm 1.49 (1.28–1.74) (Table 2).

**Table 1:** Age, height, and body mass index of study patients

Variable	Total	≤ 160 cm	161–165 cm	166–170 cm	171–175 cm	> 175 cm
*N*	135,741	28,672	35,334	39,004	21,147	21,147
Age (mean, SD)	39.8 (15.2)	43.1 (17.0)	40.6 (15.6)	39.2 (14.5)	37.2 (13.3)	35.7 (12.2)
Height (mean, SD)	166.4 (6.6)	157.4 (3.0)	163.6 (1.3)	168.4 (1.3)	173.0 (1.3)	178.6 (2.7)
BMI (mean, SD)	26.0 (5.7)	26.6 (5.8)	26.1 (5.7)	25.7 (5.7)	25.5 (5.7)	25.5 (5.7)

**Table 2:** Association between body height and BC (multivariable logistic regression)

Model	Body height	OR (95% CI)^a^	*P* value
Model 1	161–165 cm	1.13 (1.03–1.25)	0.012
	166–170 cm	1.26 (1.15–1.39)	< 0.001
	171–175 cm	1.43 (1.27–1.61)	< 0.001
	> 175 cm	1.49 (1.28–1.74)	< 0.001
		Reference	< 0.001
Model 2	Effect per 10 cm increase in height	1.18 (1.12–1.24)	< 0.001

^a^Multivariable logistic regression adjusted for age and body mass index

## Discussion

In this large retrospective study carried out in Germany, we identified a trend of increasing BC prevalence with increasing body height and found a highly significant positive association between body height and BC risk using multivariable logistic regression models adjusted for age and body mass index.

In general, the significant positive association between body height and BC risk is in line with numerous epidemiological studies published previously [15–21]. More recently, a large meta-analysis conducted by Zhang et al. was performed to investigate associations between height and BC risk using data from 159 prospective cohort studies performed in several countries (e.g., USA, Canada, Sweden, and Norway). They showed that the pooled RR for developing BC was 1.17 (95% confidence interval CI 1.15–1.19) per 10 cm increase in height. The authors also analyzed height-related genetic variants (single-nucleotide polymorphisms, SNPs) and determined that eight genetic variants were associated with an increased BC cancer risk. Further Mendelian randomization analysis reveals an odds ratio of 1.22 (95% CI 1.13–1.32) for BC per 10 cm increase in genetically predicted height and provides strong evidence that the association between adult height and BC risk is likely to be causal [22].

Although height is non-modifiable for the individual, it should be mentioned that final body height is the result of various genetic and environmental factors occurring before birth and during childhood and adulthood, and there is growing evidence that these factors (e.g., childhood diet and nutritional status) can also influence cancer risk in adulthood [23, 24]. In particular, biological pathways such as insulin-like growth factor-1 (IGF-1) signaling are involved in both adult body height and carcinogenesis and therefore considered a possible causal link regarding height and cancer risk [25, 26]. Notably, all factors of the IGF-1 system, including IGF-1, IGF-binding proteins (IGFBPs), and the IGF-1 receptor (IGF-1R) play a pivotal role in BC development, progression, and metastasis [27–29]. Large prospective population studies from many different countries have shown an increasing BC risk with increasing serum levels of IGF-1. This BC risk related to IGF-1 level was highly significant for premenopausal women only, indicating the possible importance of IGF-1 levels in early life or with respect to an influence on mammary gland development in women [30–33]. Conversely, in another prospective study it was shown that mutations in the growth hormone receptor (GHR) gene lead to reduced IGF-1 levels, which are associated with severe short stature in the subjects concerned and significantly reduced diabetes and BC risks [34]. However, the exact reason for the increased BC risk with increasing body height remains unclear and further research is necessary to uncover the underlying mechanisms.

According to the literature, analyzing the association between body height and cancer risk requires adjustments for potential confounding factors to achieve reliable results (Table 1). For instance, the association between BC risk and overweight varies according to menopausal status [35, 36]. In particular, obese women have a reduced risk of hormone receptor (HR)-positive premenopausal BC and an increased risk of HR-positive postmenopausal BC compared to women with a normal BMI, whereby the precise mechanism of these paradoxical effects remains elusive [5, 37, 38]. It seems clear that among premenopausal women, overweight leads to anovulation and lower estrogen levels while adipose tissue in obese postmenopausal women produces considerable amounts of estrogen, leading to an increased BC risk [39]. As is well-known for other diseases, age is the most important non-modifiable risk factor for BC. The BC incidence in Germany is relatively low before the age of 30 (< 50 per 100,000 women) but increases strongly until the age of 65 (300 per 100,000 women) [40, 41]. However, after adjustment for these possible confounding factors, we were able to present reliable data for an increased BC risk with increasing body height in this large retrospective study of women in Germany.

### Strengths and limitations

Our retrospective cohort study has several strengths: The German Disease Analyzer (DA) is a large European outpatient database containing data from 161 gynecological practices in Germany. The representativeness of the diagnoses it contains has already been validated [12]. Furthermore, a large patient cohort (135,741 women) was used for this study and to avoid confounding factors, adjustment for age and BMI was performed.

However, the study results should be interpreted in the light of several limitations: The DA does not contain information on external confounding factors (e.g., alcohol, tobacco consumption, socioeconomic status) and body height was only available for 16% of all patients. Moreover, the average BMI (26.0 kg/m^2^) of women within the study indicates that tendentially more overweight women are part of the study population. In addition, there is a lack of detailed information regarding the molecular subtype of BC, TNM classification, menopausal status, and other covariates such as hormone replacement therapy (HRT).

Finally, this study is a retrospective database analysis that does not allow conclusions to be drawn about causal relationships.

## Data Availability

Anonymized raw data are available on reasonable request.
